# A depth iterative illumination estimation network for low-light image enhancement based on retinex theory

**DOI:** 10.1038/s41598-023-46693-w

**Published:** 2023-11-12

**Authors:** Yongqiang Chen, Chenglin Wen, Weifeng Liu, Wei He

**Affiliations:** 1 School of Automation, Guangdong University of Petrochemical Technology, Maoming, 525000 China; 2 School of Electrical and Control Engineering, Shaanxi University of Science and Technology, Xi’an, 710021 China

**Keywords:** Computer science, Information technology

## Abstract

Existing low-light image enhancement techniques face challenges in achieving high visual quality and computational efficiency, as well as in effectively removing noise and adjusting illumination in extremely dark scenes. To address these problems, in this paper, we propose an illumination enhancement network based on Retinex theory for fast and accurate brightening of images in low-illumination scenes. Two learning-based networks are carefully constructed: decomposition network and enhancement network. The decomposition network is responsible for decomposing the low-light input image into the initial reflectance and illumination map. The enhanced network includes two sub-modules: the illumination enhancement module and the reflection denoising module, which are used for efficient brightness enhancement and accurate reflectance. Specially, we have established a cascaded iterative lighting learning process and utilized weight sharing to conduct accurate illumination estimation. Additionally, unsupervised training losses are defined to improve the generalization ability of the model. The proposed illumination enhancement framework enables noise suppression and detail preservation of the final decomposition results. To establish the efficacy and superiority of the model, on the widely applicable LOL dataset, our approach achieves a significant 9.16% increase in PSNR compared to the classical Retinex-Net, and a remarkable enhancement of 19.26% compared to the latest SCI method.

## Introduction

In recent times, the advancement of imaging technologies has allowed us to capture a plethora of visual information even in challenging low-light conditions. However, these low-light images often suffer from diminished visibility, reduced contrast, and loss of crucial details. Such limitations can hinder various applications, including surveillance, photography, and autonomous systems.To address this issue, the field of low-light image enhancement (LLIE) has emerged as a pivotal research domain, aiming to restore and enhance the perceptual quality of images captured under inadequate lighting conditions.

Presently, artificial intelligence has made remarkable strides in domains like image recognition^1^, object detection^2^, semantic segmentation^3^, and autonomous driving^4^. However, these advancements primarily pertain to well-lit daytime scenarios, with limited discourse on target recognition and detection techniques under challenging conditions such as low-light, insufficient exposure, and uneven illumination commonly encountered in nighttime settings. Due to the low brightness, poor contrast, and color distortion of images and videos captured at night, the effectiveness of visual systems such as object detection and recognition is seriously weakened. Enhancing the quality of images captured under low light conditions through LLIE can help improve the accuracy and effectiveness of many imaging-based systems. Therefore, LLIE is an essential technique in computer vision applications.

Currently, various methods have been proposed for LLIE, including histogram equalization, Non-local Means Filtering, Retinex-based methods, multi-exposure fusion, and deep learning-based methods, among others. Among them, LLIE methods based on Retinex theory have gained much attention due to their ability to simulate human visual color perception. According to Retinex theory^5^, an observed image *I* can be represented as:

1 $$\begin{aligned} I=R \otimes L \end{aligned}$$

where *R* represents the reflection component, and *L* represents the illumination component. In this way, the LLIE problem can be formulated as the estimation of the illumination component *L* under low-light conditions. Recently, numerous approaches^6–10^ that integrate Retinex theory with deep learning have been proposed for enhancing low-light images. While these approaches have achieved remarkable progress, three main challenges impede their practical deployment in real-world scenarios. Firstly, most CNN-based methods require training examples with references, which means that both low-light and normal-light images of the same visual scene need to be captured simultaneously. However, this is an extremely challenging task, which limits the availability of such training examples. Moreover, deep learning methods face difficulties in dealing with extremely low-light conditions, which require specialized techniques to achieve good performance. Finally, low-light images often suffer from high levels of noise, making it difficult to enhance them effectively. Previous studies have typically focused on addressing one of these challenges, rather than all three simultaneously.

**Figure 1:**
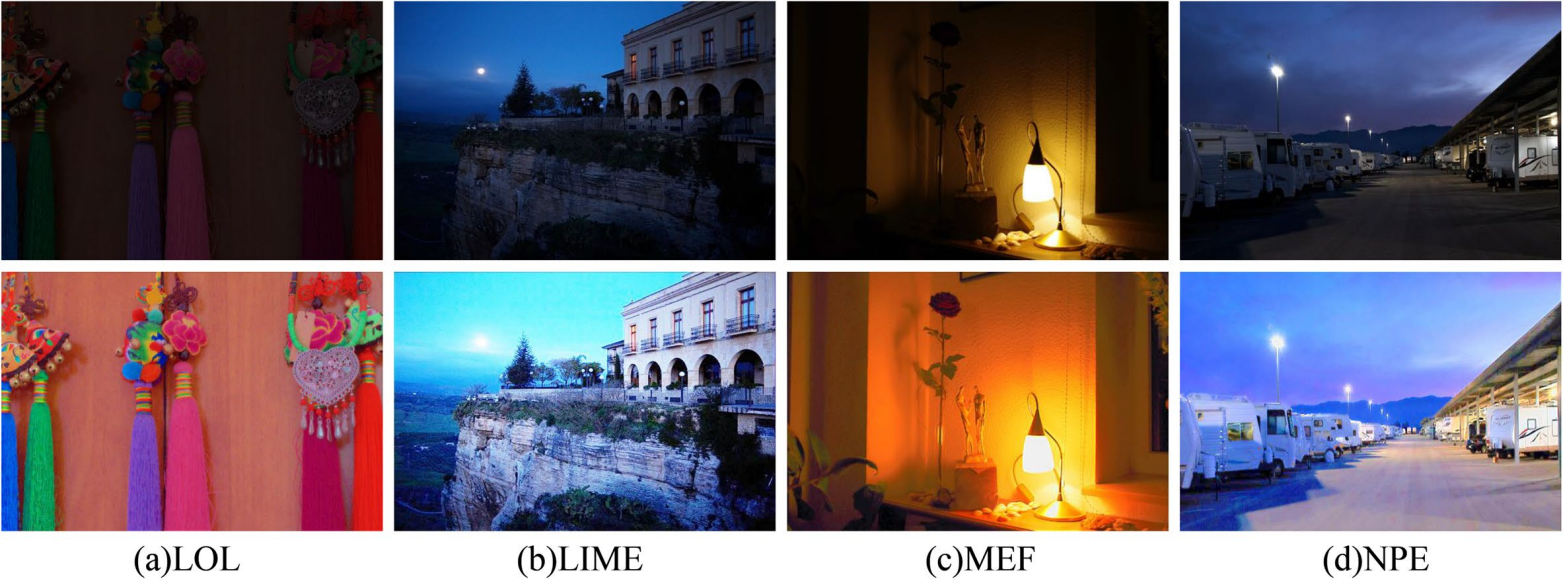
The comparison effect of various images taken in different scenes, the top represents the input low-light image and the bottom represents the enhanced image. From left to right, these real images are selected from the LOL, LIME, MEF, and NPE benchmarks, respectively.

**Our Contributions**: To address the above-mentioned challenges, we propose a data-driven deep network for learning the decomposition and enhancement of low-light images. Specifically, two separate networks are used for decomposition and enhancement tasks. The decomposition network first separates the low-light image into reflection and illumination components. Aiming at the problem of many noise points in the reflection component, the reflectance denoising module in the enhanced network is used for denoising. To address the problem of low brightness in the illumination component, an illumination enhancement module is introduced in the enhancement network, which estimates the illumination component from the low-light image in a hierarchical manner. Finally, the denoised reflection component and estimated illumination component are used for image reconstruction. Figure 1 illustrates the effectiveness of our proposed method in enhancing low-light images. Our approach enhances image brightness while preserving details, resulting in significant improvements compared to the original input image. Remarkably, some of the results are even superior to the ground truth images.

In summary, this paper makes the following main contributions:

- Based on the Retinex model, we propose a low-illumination image enhancement framework, which includes decomposition and enhancement networks corresponding to initialization, illumination map adjustment and reflection map optimization, respectively. Furthermore, the entire network is optimized with zero-shot learning instead of relying on supervised learning;
- We define unsupervised training losses to constrain the output of each stage, which enables our method to adapt to different scenes. Additionally, illumination learning with weight sharing is used to ensure convergence between the results of each stage, improve exposure stability, and significantly reduce the computational burden;
- Our method outperforms existing techniques on benchmark datasets, achieving significant improvements in evaluation metrics such as MAE, PSNR, SSIM, LPIPS (reference) and NIQE (no-reference), demonstrating its superior efficiency.

The rest of this paper is as follows: “Related works” introduces the proposed network framework. “The proposed illumination estimated network” explains the unsupervised losses used in each component. “Experimental results and analysis” presents the evaluation of our method through subjective and objective assessments on multiple datasets. Finally, “Conclusion and future work” concludes and discusses future work.

## Related works

LLIE plays an irreplaceable role in restoring intrinsic color and detail as well as compressing noise in low-light images. In the following, we provide a comprehensive review of previous work on low-light image enhancement, including both traditional and learning-based approaches.

### Model-based methods

Traditional methods for LLIE can be roughly categorized into several aspects, including Tone Mapping^11^, Gamma Correction (GC)^12^, Histogram Equalization (HE)^13^, and Retinex-based method^14^. Tone Mapping is used to create images with more details, color, and contrast, while maintaining a natural appearance. Although Tone Mapping can preserve details in images, linear mapping can result in information loss in the brightest and darkest areas. Gamma Correction performs nonlinear tone editing by editing the gamma curve of an image to detect dark and bright parts of the image signal. However, global parameters can cause local over-/under-exposure, and it is difficult to choose the value of global parameters. Rahman et al.^15^ proposed an adaptive Gamma Correction method which determines the intensity transfer function based on the statistical characteristics of the image.

Histogram equalization enhances image contrast by transforming the original image histogram into a uniform histogram, thereby expanding the dynamic range of the image. It is simple to implement and has a fast processing speed, but some local areas may not be enhanced, leading to poor performance for images with varying depths of field. Adaptive histogram equalization (AHE)^16^ is a method that has been proposed to map the histogram of local regions to a simple mathematical distribution. In contrast, Contrast-Limited Adaptive Histogram Equalization (CLAHE)^17^, which sets a threshold and assumes that if a pixel value in the histogram exceeds the threshold, the pixel is clipped, and the excess part above the threshold is uniformly distributed to every pixel. Similarly, Rahman et al.^18^ proposed the conversion of images from the RGB color space to the HUV color space. They introduced nonlinear functions to control brightness and contrast, effectively addressing the exposure issues in degraded images.

The Retinex theory is a computational theory that aims to model human visual perception of color constancy. Within this context, Dai et al.^19^ introduced a fractional-order fusion model derived from the Retinex theory. This model’s versatility extends to intricate low-light scenarios, where it proficiently heightens visual quality while simultaneously preserving intricate image details. Notably, various algorithms dedicated to color constancy, such as single-scale Retinex (SSR)^20^, multi-scale Retinex (MSR)^21^, color restoration-based Retinex (MSRCR)^22^, and white balance methods^23,24^, have been proposed. They not only achieve color constancy, but most algorithms also allow for color enhancement and high dynamic range compression of images. However, there is still room for improvement in the visual color information processing mechanism and algorithm universality of these methods. In most cases, traditional model-based approaches rely heavily on manually designed prior or statistical models. When applied to different scenarios, their effectiveness may vary.

### Deep learning-based methods

In recent years, there has been a shift towards deep learning-based approaches in the research on low-light image enhancement. LLNet^25^ is a pioneering work by LLIE that performs contrast enhancement and denoising based on a depth autoencoder. However, the relationship between real-world illumination and noise is not touched, and thus residual noise and over-smoothing problems occur. Chen et al.^6^ proposed Retinex-Net, which decomposes the input image into a reflectance map and an illumination map, and enhances the illumination map for low-light enhancement using a deep neural network. Post-processing with BM3D is then used for denoising. Although Retinex-Net is effective in enhancing the brightness and detail features of the image, the image smoothing is poor and the color distortion is severe. Lv et al.^26^ proposed an end-to-end multi-branch enhancement network (MBLLEN) consisting of feature extraction, enhancement, and fusion modules to facilitate the performance of LLIE. Inspired by the field of super-resolution reconstruction, UTVNet^27^ and URetinex^28^ proposed an adaptive unfolding network to robustly denoise and enhance low-light images.

Recently, zero-shot learning based approaches have gained attention for their efficiency and economy, requiring fewer images compared to traditional supervised learning-based approaches in LLIE. For example, Liu et al.^29^ proposed a Retinex-based Unrolling with Architecture Search (RUAS) and designed a collaborative reference-free learning strategy to discover low-light prior architectures from a compact search space. Guo et al.^30^ proposed a Zero-DCE, which is implemented through an intuitive nonlinear curve mapping. Subsequently, they developed and demonstrated a faster and more lightweight network known as Zero-DCE++^31^. Zero-DCE relies on the use of multiple exposure training data and does not account for noise while being less effective for extreme cases of enhancement. Zhu et al.^32^ introduced RRDNet, a three-branch convolutional neural network for restoring underexposed images. RRDNet utilizes an iterative approach to decompose the input image into its constituent parts: illumination, reflectance, and noise. This is achieved by minimizing a customized loss function and adjusting the illumination map through gamma correction. The reconstructed reflectance and the adjusted illumination map are then multiplied element-wise to generate the enhanced output image. Ma et al.^33^ proposed a learning framework called self-calibrating illumination (SCI) for fast and flexible enhancement in real-world low-illumination scene images. The method estimates a convergent illuminance map through the network, and based on Retinex theory, the input low-illuminance image is divided element by element with the estimated illuminance map to derive an enhanced reflectance map. Although SCI converges the illuminance map in iterations, it does not take into account the interference caused by the noise in it.

In summary, both learning-based and traditional methods have their limitations in the realm of image enhancement. Learning-based approaches often excel in adapting to complex data distributions but require substantial amounts of data and computational resources. Traditional methods might rely more on prior knowledge and are constrained by their limited non-linear modeling capabilities. In practical applications, the selection of an appropriate method is crucial, dependent on the task’s nature and available resources. Future research might delve into strategies that fuse these two approaches to overcome their respective constraints and achieve enhanced image enhancement effects.

## The proposed illumination estimated network

In the third section, we introduce our novel method for enhancing low-light images and present the detailed framework for LLIE.

**Figure 2:**
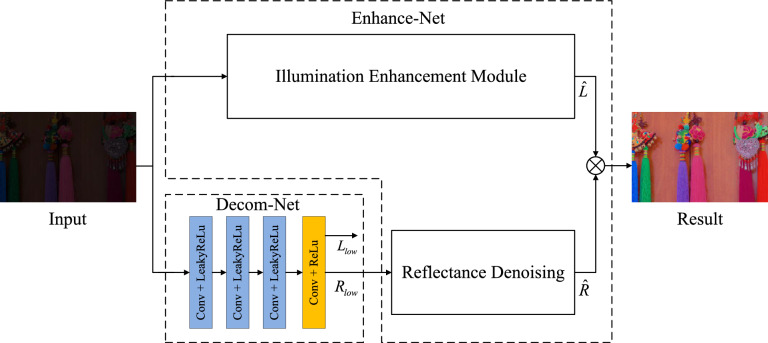
The framework of the proposed model.

As shown in Fig. 2, the proposed framework includes two networks, ie. decomposition network and enhancement network. The decomposition module decomposes the input low-illumination image *I* into illumination map $L_{low}$ and reflection map $R_{low}$ ; the illumination enhancement module estimates the appropriate illumination map $\hat{L}$ by network iteration; the reflectance denoiseing module then denoises and fine-tunes the reflection map $R_{low}$ obtained from the decomposition module to obtain the estimated reflection map $\hat{R}$; finally, according to the Retinex theory, the reflection map $\hat{R}$ is multiplied element by element with the illumination map $\hat{L}$ to obtain the enhancement result $\hat{I}_{result}$.

### Decomposition module

The inspiration for our decomposition module comes from the Retinex theory, which decomposes an image *I* into its illumination component *L* and reflection component *R*. The reflection component *R* is a constant part determined by the inherent properties of the object, while the illumination component *L* is influenced by external illumination and can be enhanced by removing the illumination influence or correcting the illumination component. The classic Retinex-Net proposed by Chen et al.^6^ introduces a large amount of random noise and color shifts when decomposing the input image, so it is crucial to choose an appropriate decomposition strategy for image enhancement.

To obtain more informative initial illumination and reflectance without introducing distortion, we propose a data-dependent decomposition module called Decom-Net. This module uses a fully convolutional network to simultaneously learn $R_{low}$ and $L_{low}$ in an adaptive manner. The Decom-Net module consists of three Conv+LeakyReLU layers and one convolutional layer followed by a ReLU layer, with a kernel size of $3\times 3$ for all convolutional layers. The first three Conv+LeakyReLU layers capture low-level features while suppressing noise and extraneous information. The subsequent convolution and ReLU layers refine the decomposition results to produce the final illumination and reflection components. By incorporating the Decom-Net module, we aim to uncover coarse details in a data-dependent manner while minimizing distortion.

### Illumination enhancement module

**Figure 3:**
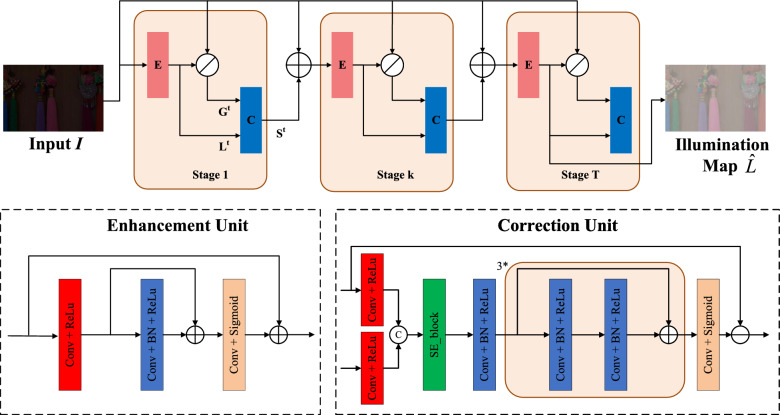
Illustration of our proposed illumination enhancement module (IAM). The IAM consists of two units: enhancement unit (E) and correction unit (C). The IAM iteratively refines the illumination layer to obtain an estimated illumination map $\hat{L}$.

Our proposed Illumination Enhancement Module (IEM) is illustrated in Fig. 3, which uses a fully convolutional network to adaptively learn the illumination estimation map $L^t$ and iteratively converge to obtain the estimated result $L^t$ of the desired illumination map. It consists of two parts: an enhancement unit and a correction unit. The original low-light input image is added to the enhancement unit to obtain a rough illumination estimation map $L^t$, which is used as the input of the correction unit in the next stage; The input of the correction unit is composed of the illumination estimation map $L^t$ obtained from the enhancement unit and the guidance map $G^t$, which are concatenated and sent into the correction unit through an attention mechanism. The output of the correction unit is denoted as $S^t$. The transformation input item obtained by adding the input image *I* with $S^t$ is used as the input of the next stage enhancement unit and participates in the overall loop. It should be noted that both units share parameters throughout the entire training process, and an accurate illumination estimation map is obtained as the final result.

#### Enhancement unit

The enhancement unit consists of three parts: the starting layer Conv+ReLU, the middle layer Conv+BatchNorm+ReLU, and finally the Conv+Sigmoid layer. The convolutional kernel size is uniformly set to $3\times 3$, and the dilatation rate is set to 1 so that it expands the perceptual field of the convolutional network without increasing the computational burden and improves the ability to extract features. the BatchNorm layer normalizes each channel to reduce the dependency between channels, and at the same time accelerates the convergence of the network. The enhancement unit initially gets the estimated light component $L^t$ in preparation for the next stage. The enhancement unit is denoted as:

2 $$\begin{aligned} \begin{aligned} \mathscr {F}\left( {{L}^{t}} \right) : {L}^{t+1}=\mathscr {H}_{\theta }\left( {{L}^{t}} \right) , {{L}^{0}}=I \end{aligned} \end{aligned}$$

Where *L* represents the illumination term and *t*-th stage ($t = 0,\ldots ,T-1$) represents the different stages and each stage uses the same network architecture $\mathscr {H}$ and weight $\theta$.

#### Correction unit

The correction unit is designed to ensure that the results of each stage converge to the same state. The input for each stage comes from the previous stage, with the first stage taking a low-light image *I* as input. To achieve convergent, a correction image *S* is introduced and added to the low-light image, displaying the difference between each stage and the input of the first stage. This indirectly explores the convergence behavior between each stage, ensuring that the results are almost the same in all stages.

The correction unit is similar to the enhancement unit, consisting of five parts: the initial layer of Conv+ReLU, an attention block, followed by a layer of Conv+BatchNorm+ReLU, 3 stacked identical network structures composed of Conv+BatchNorm+ReLU, and a final layer of Conv+Sigmoid to ensure the output is within the range of 0–1. The size of the convolution kernel is also set to $3\times 3$, and the dilation rate is set to 1. First, the input *I* is divided element-wise by the illumination map $L^t$ to obtain the guidance map $G^t$. Then, the input to the correction unit consists of the illumination estimation image $L^t$ obtained from the enhancement unit and the guidance image $G^t$. However, the degree of distortion in the guidance image *G* is highly dependent on the brightness of the illumination layer *L*, with darker areas exhibiting more severe degradation. Therefore, these two inputs are concatenated and fed into the correction unit $\mathscr {K}_{\theta }$ as input, guided by an attention mechanism^34^ to guide the recovery of the correction image *S*. Finally, the input $I^t$ is added to the correction image $S^t$ to obtain the converted term $V^t$, which is the input to the next stage of the enhancement unit.

3 $$\begin{aligned} \mathscr {G}\left( L^t\right) :\left\{ \begin{array}{l} G^t = I \oslash L^t \\ S^t = \mathscr {K}_{\theta }\left( G^t, L^t\right) \\ V^t = I+S^t \end{array}\right. \end{aligned}$$

The iterative convergence of illumination *L* is expressed as:

4 $$\begin{aligned} \mathscr {F}\left( {{L}^{t}} \right) \rightarrow \mathscr {F}\left( \mathscr {G}\left( {{L}^{t}} \right) \right) \ \end{aligned}$$

In the training phase, *T* is set to 3 to ensure that $L^t$ tends to be consistent in the iteration. At the same time, to reduce the computational burden and improve the image processing speed, only one enhancement unit is selected in the test phase to obtain the enhanced illumination image $\hat{L}$ from input *I*.

### Reflectance denoising module

**Figure 4:**
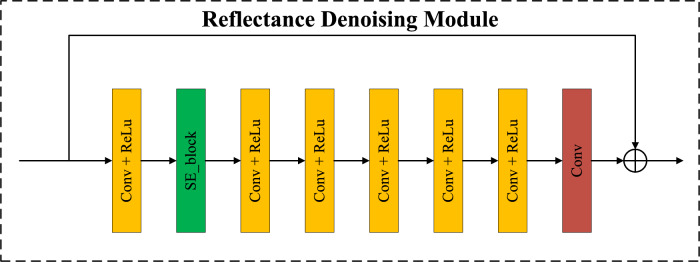
Illustration of our proposed reflectance denoising module (RDM).

Due to factors such as the collection environment and equipment, low-light images often contain a large amount of noise in dark areas. Noise reduces image information and lowers image quality. At the same time, the reflection component reflects object features and contains a large amount of detailed information. To better process low-light images, it is necessary to achieve good noise reduction and detail preservation effects. As shown in Fig. 4, an improved Reflectance Denoising Module (RDM) is used to denoise the reflection component $R_{low}$ of the low-light image.

The RDM aims to solve the clear reflection component *R*. The initial reflection component $R_{low}$ containing noise obtained from the decomposition module is fine-tuned to obtain the denoised reflection component. It consists of a SE block, six Conv+ReLU layers, and one Conv layer. The convolution kernel size is uniformly set to $3\times 3$ across the entire convolutional layer, and the padding mode is set to replicate to prevent edge artifacts. By using a learnable denoising network $\mathscr {G}\left( R\right)$ in a similar way to DnCNN^10^, the degraded $R^t$ can be transformed into a cleaner reflection map. Thus, the network used to perform the *R* update is represented as:

5 $$\begin{aligned} \mathscr {G}\left( {{R}^{t}} \right) \rightarrow \mathscr {G}\left( {{R}^{t+1}} \right) \ \end{aligned}$$

This module uses a residual learning strategy to remove the same part of the main part of the image so that the model focuses on learning small changes in the image, so that the network directly learns the residual image, that is, the difference between the noisy image and the clear image. This method helps the model to focus on learning the noise in the image and indirectly obtain a clear image by subtracting the learned residual image from the noise image. The denoised reflectance and enhanced illumination are then combined into the final result through element-wise multiplication, which can be expressed as: $\hat{I}_{result} = \hat{R} \otimes \hat{L}$.

**Figure 5:**
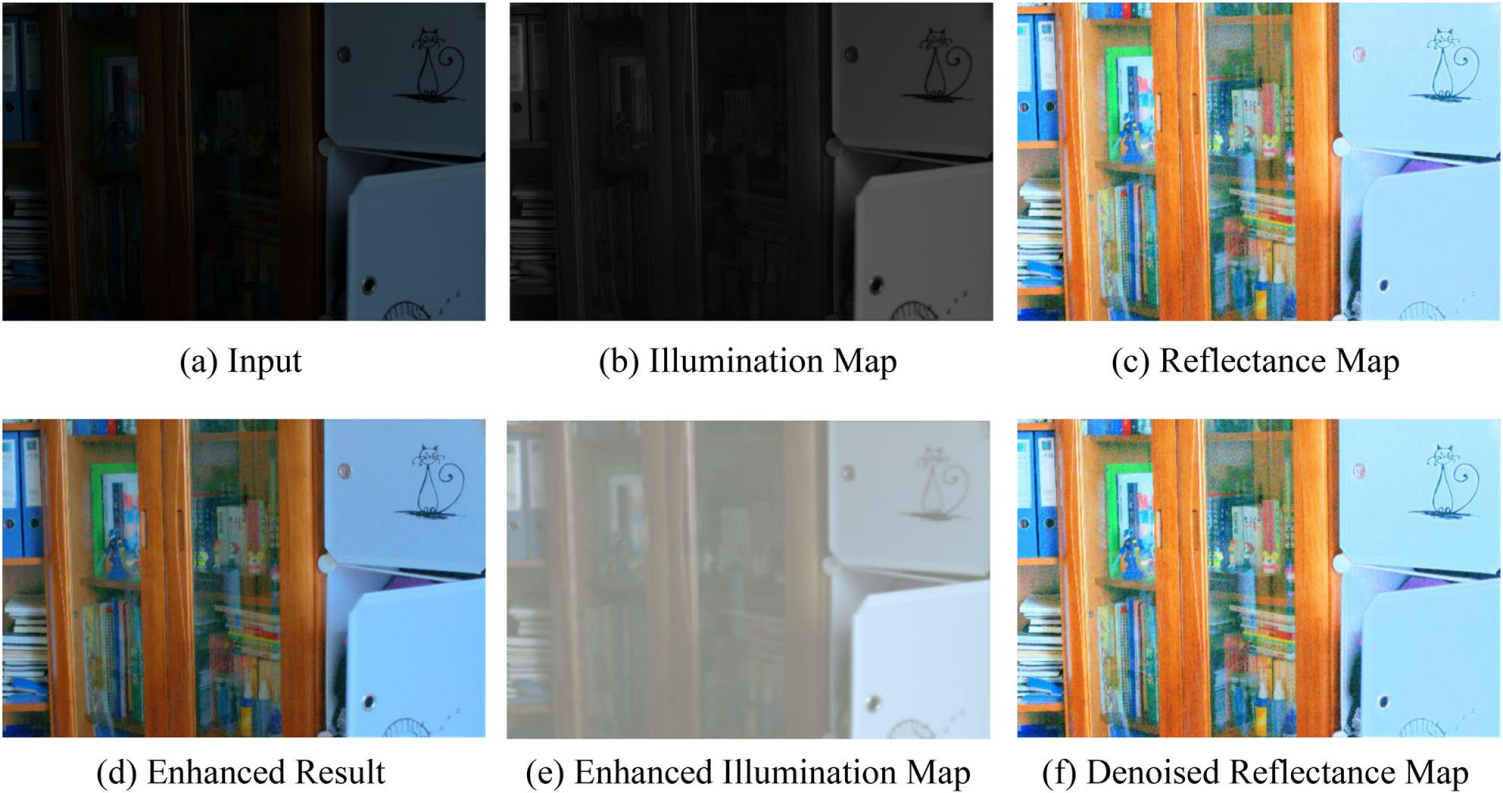
The decomposition results obtained by our method. The illumination map and reflectance map are obtained by the Decom-Net, the enhanced illumination map is obtained by IAM, and the denoised reflectance map is obtained by RDM.

Figure 5 provides a detailed illustration of the results obtained by our method in different modules. Specifically, the reflectance map obtained by our Decom-Net module effectively preserves the original image details while suppressing noise. Meanwhile, the illumination map obtained by our illumination enhancement module (IEM) significantly improves contrast and retains more details.

### Unsupervised loss function

In the training phase, the two subnets are trained separately, so the whole loss function consists of two parts: the decomposition loss $L_{Dc}$ and the enhancement loss $L_{En}$. These loss functions are described in detail as follows.

**Decom-Net Loss**: To initialize the two components of low-light images, the loss function is designed as follows:

6 $$\begin{aligned} \mathscr {L}_{Dc}=\left\| I-R_{low} \cdot L_{low}\right\| _1+\mu \left\| L_{low}-\max _{c \in \{R, G, B\}} I^{(c)}\right\| _F^2 \end{aligned}$$

The expression $\left| |\cdot \right| |_1$ represents the *L*1 norm, while $\mu =0.1$ is a hyperparameter, and $c\in \{R, G, B\}$ refers to the RGB color channels. The first term of the loss function is intended to measure the reconstruction error, while the second term is devised to promote the preservation of the overall structure of the input image by the initialized illumination.

**Enhance-Net Loss**: We utilize a combined loss function for both reflectance and illumination, comprising of several components, namely the fidelity loss, smooth loss, reflection consistency loss, and total variation loss.

7 $$\begin{aligned} \mathscr {L}_{En}=\omega _{f}\mathscr {L}_{f}+\omega _{s}\mathscr {L}_{smooth}+\omega _{rc}\mathscr {L}_{rc}+\omega _{TV}\mathscr {L}_{TV} \end{aligned}$$

The fidelity loss is a constraint that ensures the preservation of pixel-level consistency between the input of each stage and the estimated illumination, expressed mathematically as:

8 $$\begin{aligned} \mathscr {L}_f=\sum _{t=1}^T\left\| L^t-\left( I+S^{t-1}\right) \right\| ^2 \end{aligned}$$

where *T* denotes the total number of stages.

Smooth loss: Mainly to measure the degree of smoothing between the enhanced image and the real image, to avoid the loss of the original image structure. Smooth loss is a type of regularization technique used in deep learning to encourage the model to produce smoother predictions by penalizing abrupt changes in the output. It is presented as:

9 $$\begin{aligned} \mathscr {L}_{smooth}=\sum _{i=1}^N \sum _{j \in \mathscr {N}(i)} w_{i, j}\left| L_i^t-L_j^t\right| \end{aligned}$$

Where *N* is the total number of pixels, *i* represents the *i*-th pixel, and $\mathscr {N}(i)$ represents the neighboring pixels of *i* in its 5x5 window. $w_{i, j}$ denote the weights, and the equation is of the form:

10 $$\begin{aligned} \begin{aligned} {w_{i, j}=\exp \left( -\frac{\sum _c\left( \left( I_{i, c}+S_{i, c}^{t-1}\right) -\left( I_{j, c}+S_{j, c}^{t-1}\right) \right) ^2}{2 \sigma ^2}\right) } \end{aligned} \end{aligned}$$

The symbol *c* represents the image channel in the YUV color space, while $\sigma$ = 0.1 denotes the standard deviation of the Gaussian kernel.

Reflection consistency loss: To maintain the inherent characteristics of the image according to the Retinex theory, the reflection component should remain unchanged during image processing. To ensure this, the reflection consistency loss function is utilized in this paper, which constrains the consistency of the reflection between the input and output images. The function can be expressed as:

11 $$\begin{aligned} \mathscr {L}_{rc}=\sum _{t=1}^T\left\| R_{low}-R^t\right\| ^2 \end{aligned}$$

Total Variation loss: To suppress noise, we use total variation to further refine the image $R^t$. While the TV loss helps reduce noise, it may also cause a blur effect on the image structure. Thus, we set its weight to a lower value.

12 $$\begin{aligned} \mathscr {L}_{TV}=\sum _{t=1}^T\left\| \nabla \left( R^t\cdot L^t\right) \right\| _1 \end{aligned}$$

In the experiment, Empirically the four weights are set to 2 1 1 0.1.

## Experimental results and analysis

In this part, we describe the experimental results and analysis in detail. First, we briefly introduce the parameter setting and comparison methods. Then, the qualitative and quantitative evaluation of paired and unpaired data sets is described. Finally, the experimental results are analyzed.

### Experimental settings

In the following section, we provide detailed information on our parameter settings, comparison methods, and evaluation index.

For all experiments in this paper, we maintained a uniform configuration environment, which included an Ubuntu system with 32 GB RAM and an NVIDIA GeForce RTX3090 GPU. The network framework was constructed using PyTorch and optimized by ADMM with the following parameters: $\beta _1=0.9$, $\beta _2=0.99$, and $\epsilon =0.95$. The batch size was set to 16, while the learning rate was set at 0.0003. Additionally, the training sample size was uniformly adjusted to 320 $\times$ 320, and we used 485 paired images randomly selected from the LOL dataset to train our model. The training epoch number was set to 1000.

To evaluate the performance of our proposed network on low-light image datasets, we conducted a visual analysis and compared it with other state-of-the-art methods and codes. The traditional methods including HE^13^ and Tone Mapping^11^, supervised methods such as Retinex-Net^6^, unsupervised methods including RUAS^29^, Zero-DCE^30^, SCI^33^, and RRDNet^32^ were used for comparison. We selected two paired data sets (LOL and LSRW) and three unpaired data sets (LIME, MEF, and NPE) for verification experiments to test their performance in image enhancement.

For quantitative evaluation, we use mean absolute error(MAE), peak signal-to-noise ratio(PSNR), structural similarity index (SSIM)^35^, learned perceptual image patch similarity(LPIPS)^36^ and natural image quality evaluator(NIQE)^37^ as metrics. The following content provides the explicit definitions of these five evaluation metrics.

MAE: The MAE is calculated using the sum of the absolute values of the grayscale differences between the evaluation image and the original image at each point divided by the size of the image. The smaller the value indicates the smaller the deviation from the original image and the better the image quality.

13 $$\begin{aligned} \begin{aligned} MAE=\frac{1}{M\times N}\underset{i=1}{\overset{M}{\mathop {\sum }}}\,\underset{j=1}{\overset{N}{\mathop {\sum }}}\,\left| g(x,y)-\hat{g}(x,y) \right| \end{aligned} \end{aligned}$$

*M* and *N* denote the number of pixel points in the length and width of the image, respectively, *g*(*x*, *y*)and $\hat{g}(x,y)$ are the gray-scale values at the points of the original image and the image to be evaluated, respectively.

PSNR: PSNR is a widely used image quality metric that measures the difference between two images based on their pixel-level differences. It is a full-reference metric, which means that it requires a reference image to evaluate the quality of a distorted or compressed image. Mathematically, The PSNR formula can be expressed as:

14 $$\begin{aligned} \begin{aligned} PSNR=10\times \log \frac{I_{max}^{2}}{MSE} \end{aligned} \end{aligned}$$

where *MSE* is the mean square error between images and $I_{max}$ is the maxi-mum pixel value of the two images. its definition is:

15 $$\begin{aligned} MSE=\frac{1}{M\times N}\underset{i=1}{\overset{M}{\mathop {\sum }}}\,\underset{j=1}{\overset{N}{\mathop {\sum }}}\,{{[g(x,y)-\hat{g}(x,y)]}^{2}} \end{aligned}$$

SSIM: SSIM is a useful metric for evaluating image quality in scenarios where a reference image is available and the perceptual features of the image are important. It is used to highlight differences in brightness, contrast, and structural similarity between two images. Its values range from 0 to 1, where values closer to 1 indicate a higher degree of similarity between the two images. Assuming x and y are two input images, the formula is:

16 $$\begin{aligned} SSIM={{[l(x,y)]}^{\alpha }}{{[c(x,y)]}^{\beta }}{{[s(x,y)]}^{\gamma }} \end{aligned}$$

where *l*(*x*, *y*) is the brightness comparison, *c*(*x*, *y*) is the contrast comparison, *s*(*x*, *y*) is the structure comparison. $\alpha$, $\beta$, $\gamma$ is greater than 0, is used to adjust the proportion of three parts. *l*(*x*, *y*), *c*(*x*, *y*) and *s*(*x*, *y*) are the following equations, respectively.

17 $$\begin{aligned} l(x,y)=\frac{2{{\mu }_{x}}{{\mu }_{y}}+{{c}_{1}}}{\mu _{x}^{2}+\mu _{y}^{2}+{{c}_{1}}},\quad c(x,y)=\frac{2{{\sigma }_{xy}}+{{c}_{2}}}{\sigma _{x}^{2}+\sigma _{y}^{2}+{{c}_{2}}},\quad s(x,y)=\frac{{{\sigma }_{xy}}+{{c}_{3}}}{{{\sigma }_{x}}{{\sigma }_{y}}+{{c}_{3}}} \end{aligned}$$

where $\mu _x$ and $\mu _y$ denote the mean of the two images, $\sigma _x$ and $\sigma _y$ denote the standard deviation of the two images, respectively. $\sigma _{xy}$ denotes the covariance of the two images. $c_1$, $c_2$ and $c_3$ serve to avoid the denominator being zero.

LPIPS: LPIPS is a deep learning based image quality assessment metric. For LPIPS, we use an AlexNet-based model to calculate perceptual similarity. the lower the LPIPS value, the closer the result is to its corresponding ground truth in terms of perceptual similarity.

Given a ground truth image reference block *x* and a noise-containing image distortion block $x_0$, the perceptual similarity measure is formulated as follows:

18 $$\begin{aligned} \begin{aligned} d\left( x, x_0\right) =\sum _l \frac{1}{H_l W_l} \sum _{h, w}\left\| w_l \odot \left( \hat{y}_{h w}^l-\hat{y}_{0 h w}^l\right) \right\| _2^2 \end{aligned} \end{aligned}$$

where *d* is the distance between *x* and $x_0$. The feature stack $\hat{y}_{h w}^l$ and $\hat{y}_{0 h w}^l$ are extracted from the *L* layer and unit-normalized in the channel dimension. Using vectors $w_l$ to deflate the number of activated channels, the $l_2$ distance is finally calculated. Finally, it is averaged over the space and summed over the channels.

NIQE: NIQE is a no-reference image quality metric that measures the perceptual quality of natural images. It is based on the hypothesis that natural images have certain statistical properties that are correlated with their perceptual quality. These properties include texture richness, edge sharpness, and colorfulness, among others. The NIQE index is calculated mainly by calculating the distance between the input image and the Multivariate Gaussian Model (MVG) of the natural image, and the lower the value of NIQE, the better the quality of the image. Mathematically, NIQE formula is :

19 $$\begin{aligned} NIQE=D\left( v_1, v_2, m_1, m_2\right) =\sqrt{\left( \left( v_1-v_2\right) ^T\left( \frac{m_1+m_2}{2}\right) ^{-1}\left( v_1-v_2\right) \right) } \end{aligned}$$

where $v_1$, $v_2$, $m_1$ and $m_2$ represent the average vector and covariance matrix of the natural MVG model and the distorted image MVG model, respectively.

By using diverse datasets and evaluating multiple metrics, we obtained a comprehensive evaluation of our algorithm’s performance in enhancing low-light images across various scenarios.

### Subjective visual evaluation

**Figure 6:**
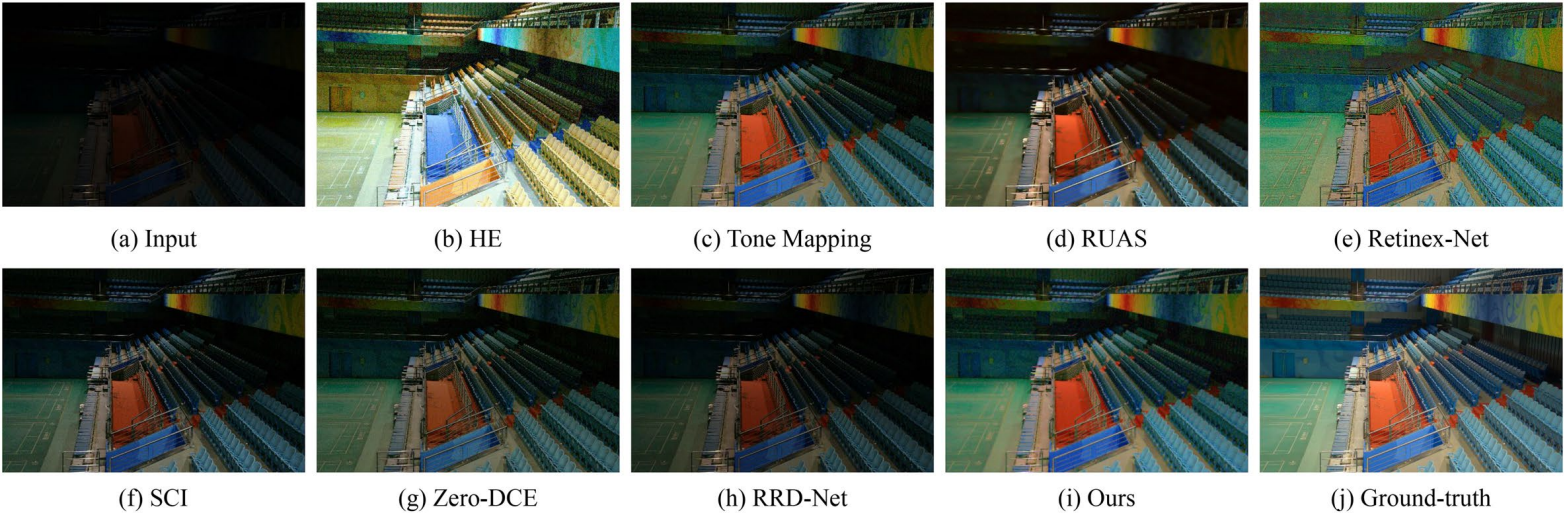
Visual comparisons of different approaches on the LOL benchmark.

**Figure 7:**
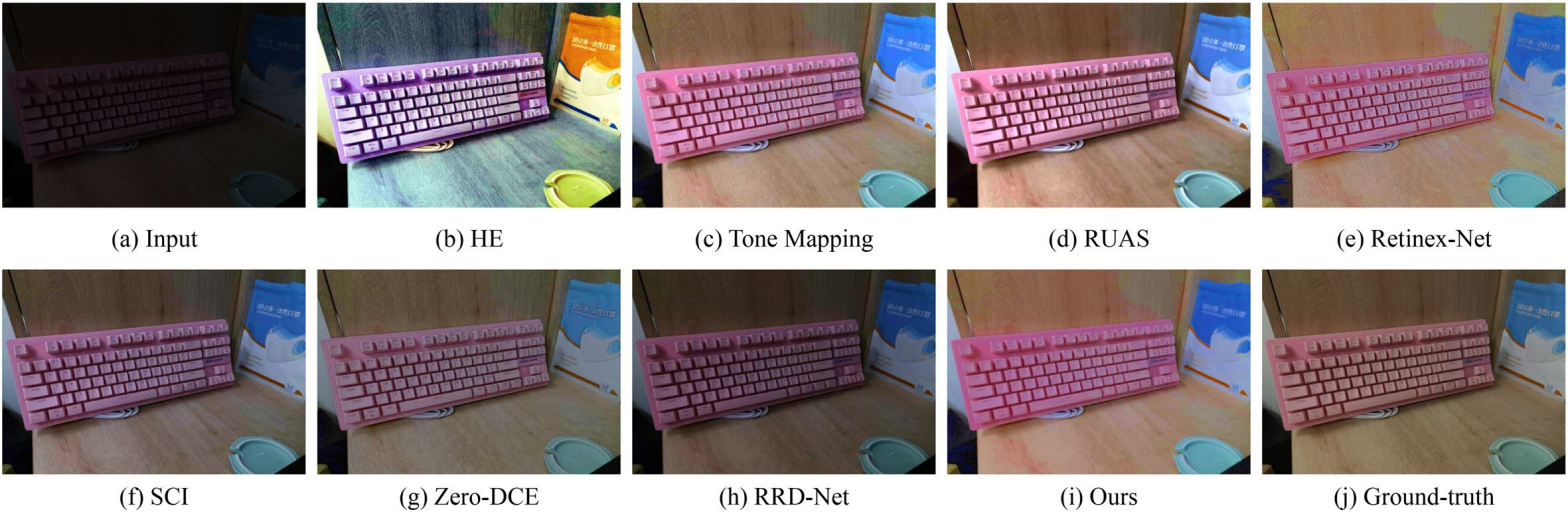
Visual comparisons of different approaches on the LSRW benchmark.

Figures 6 and 7 show some representative results of visual comparison of various algorithms. Figures 6 and 7 belong to the LOL and LSRW datasets, respectively. In Fig. 6, the enhanced results show that HE can significantly increase the brightness of low-light images. However, it applies contrast enhancement to each channel of RGB separately, causing color distortion. Retinex-Net significantly improves the visual quality of low-light images, but it overly smooths out details, enlarges noise, and even causes color deviation. Tone Mapping can stretch the dynamic range of the image, but it still has an insufficient enhancement for the grandstand seating section in the image. Although the image effect of RUAS is delicate and has no obvious noise interference, it does not successfully brighten the image in extremely dark areas (such as the central seat part). SCI and RRD-Net perform poorly in darker images and cannot effectively enhance low-light images. Zero-DCE can preserve the details of the image relatively completely, but the brightness enhancement is not obvious, and the color contrast of the image is significantly reduced. From Fig. 7, it can be seen that HE has obvious image distortion and color distortion; Retinex-Net amplifies inherent noise, losing image details; SCI, Zero-DCE, and RRD-Net have weak brightness enhancement capabilities; Tone Mapping, RUAS, and our method perform extremely well in brightness and color aspects. Compared to the Ground Truth, our approach not only significantly enhances image brightness but also effectively preserves the colors and intricate details of the images to a considerable extent, thereby effectively improving the overall image quality. This accomplishment can be attributed to the inherent mechanism of our model. Our model employs a multi-stage strategy to achieve brightness adjustment, enabling robustly estimating appropriate illumination map. Concurrently, we optimize the reflectance map to enhance image details and contrast. By thoughtfully integrating these two components, we ensure that the resulting enhanced images not only exhibit improved visibility but also faithfully reproduce the characteristics of the original scenes.

**Figure 8:**
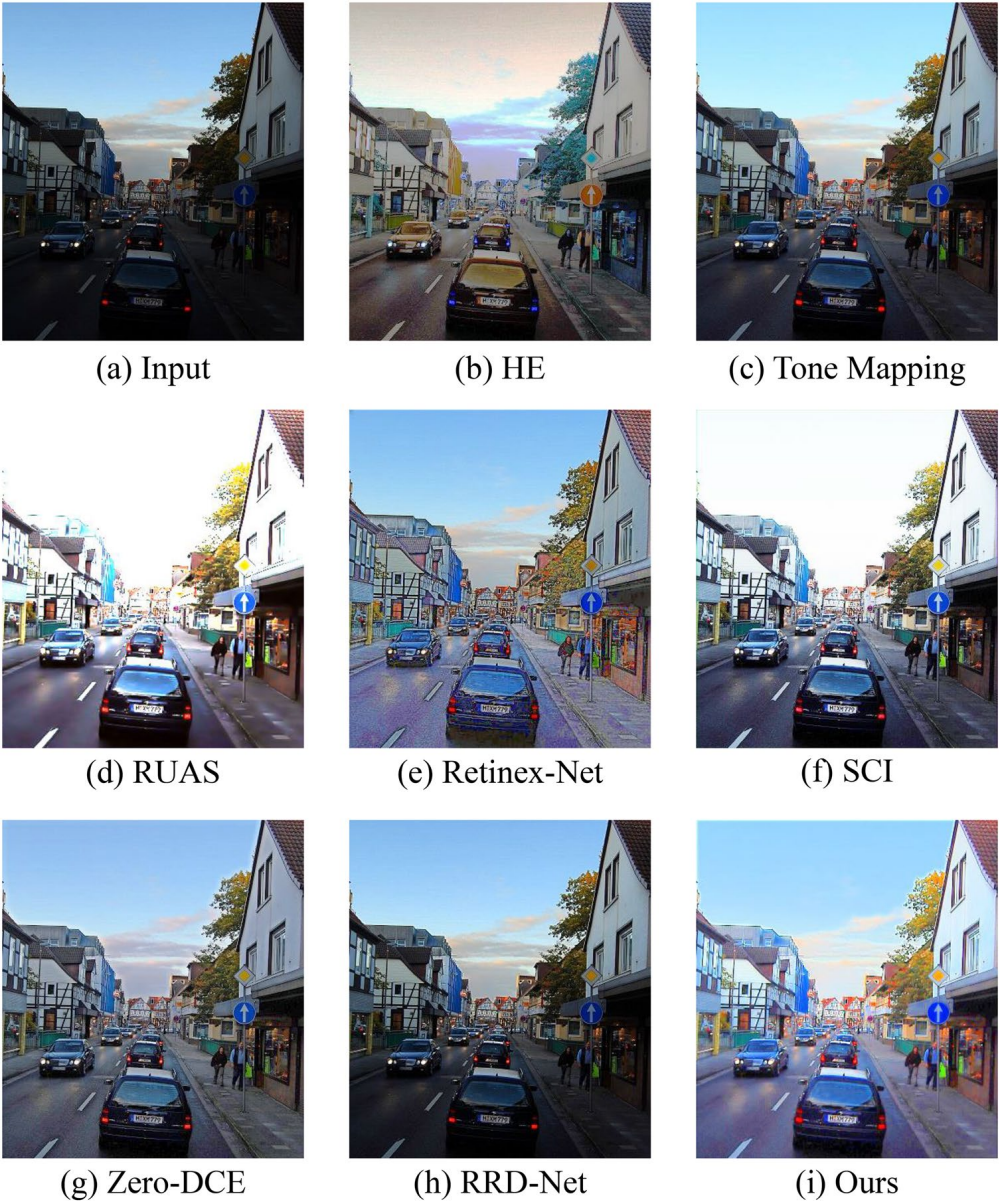
Visual comparisons of different approaches on the LIME benchmark.

**Figure 9:**
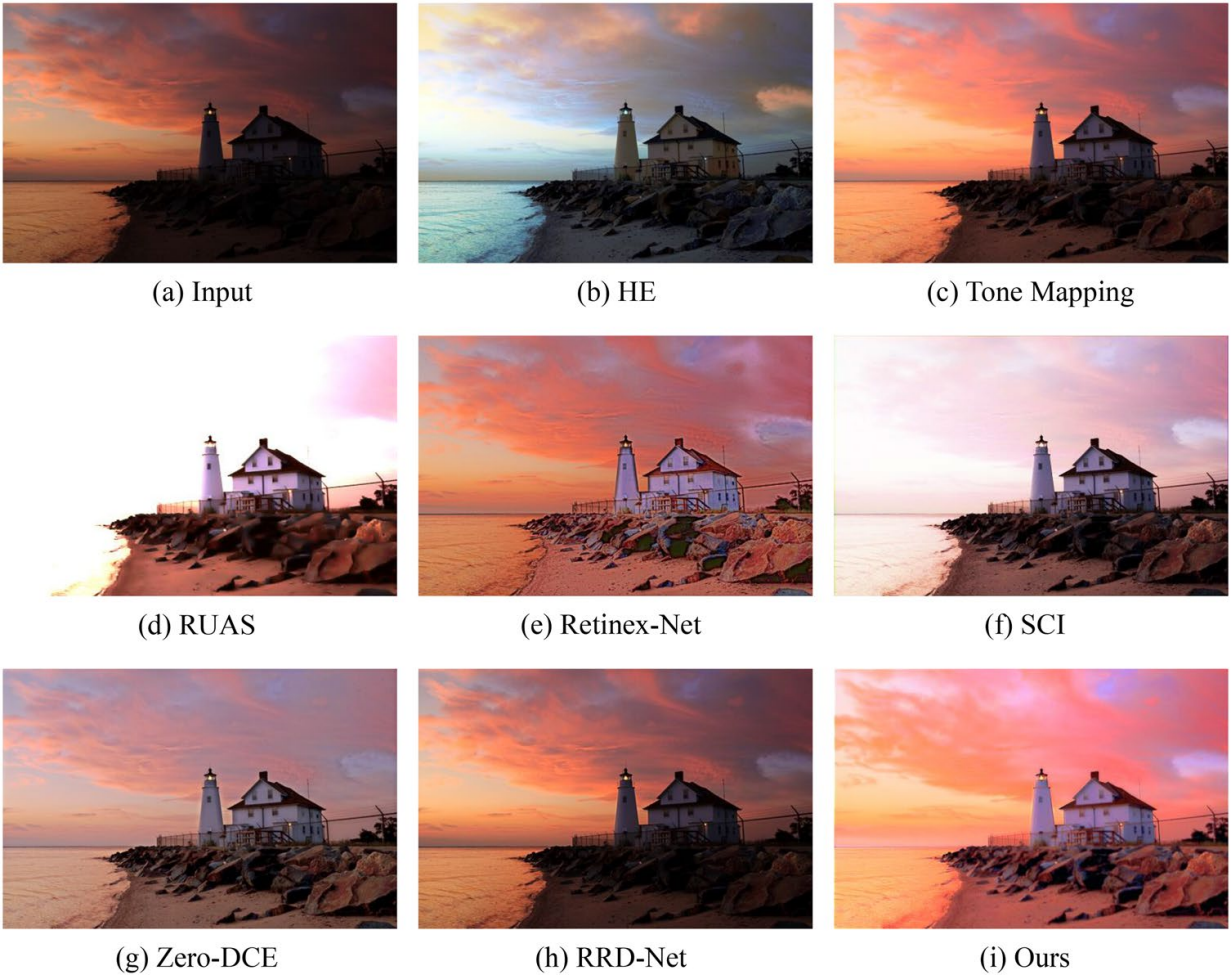
Visual comparisons of different approaches on the MEF benchmark.

**Figure 10:**
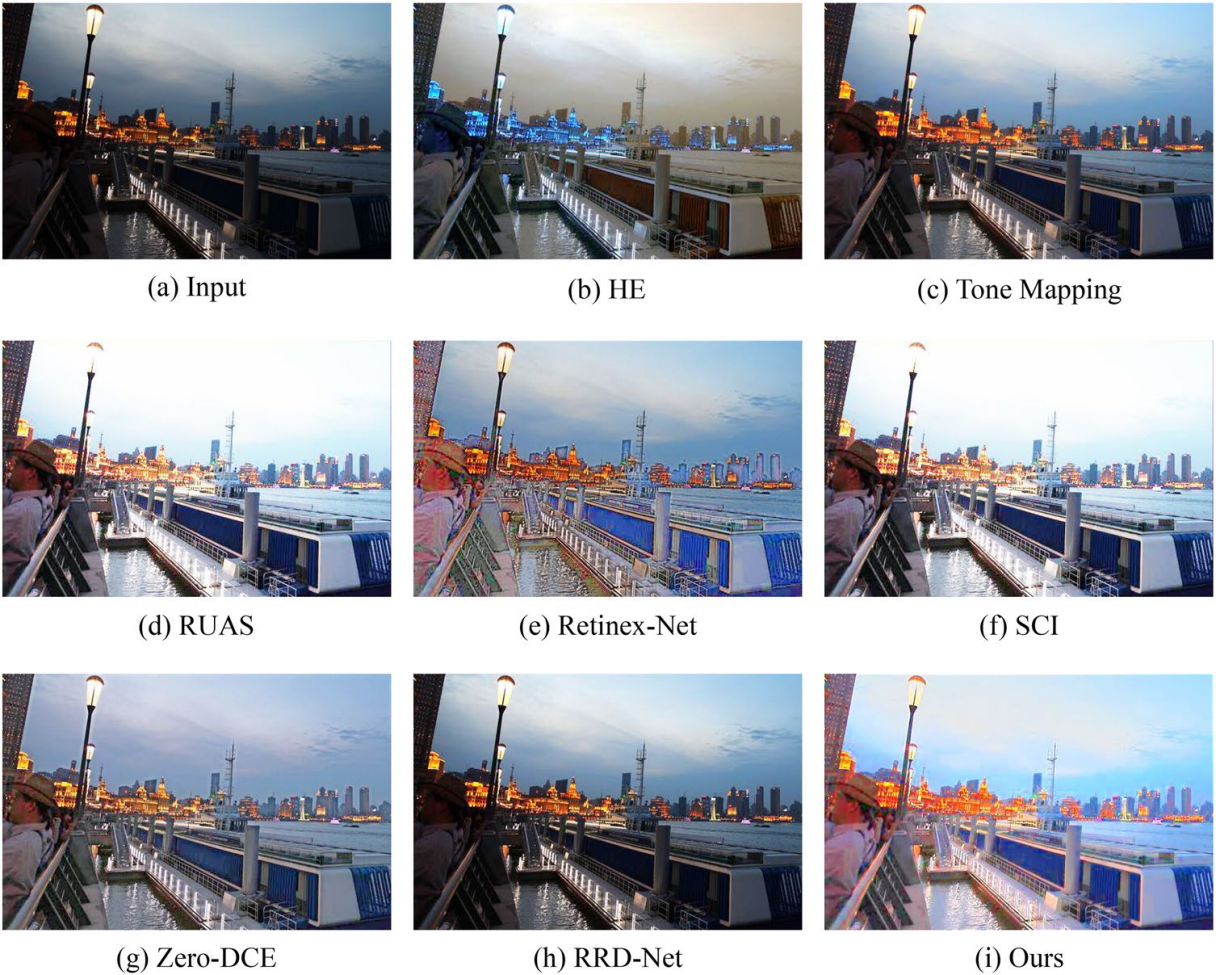
Visual comparisons of different approaches on the NPE benchmark.

To comprehensively evaluate various algorithms, we also selected three unpaired benchmarks (LIME, MEF, NPE) for verification experiments. In Figs. 8, 9 and 10, we show the visual contrast effects produced by these cutting-edge methods under various benchmarks. From these enhancement results, it can be seen that HE greatly improves the contrast of the image, but there is also a significant color shift phenomenon. Retinex-Net introduces visually unsatisfactory artifacts and noise. Tone Mapping and RRD-Net can preserve image details, but the overall enhancement strength is not significant, and they fail to effectively enhance local dark areas. RUAS and SCI can effectively enhance low-contrast images, but during the enhancement process, they tend to excessively enhance originally bright areas, such as the sky and clouds in Figs. 8, 9, and 10, which are replaced by an overly enhanced whitish tone. Among all the methods, Zero-DCE and our proposed method perform well on these three benchmarks, effectively enhancing image contrast while maintaining color balance and detail clarity.

### Objective evaluation

In addition to the subjective visual evaluation, the effectiveness of the algorithms in this paper is illustrated using the recognition image quality metric used for quantitative comparison.

In this study, we have evaluated the proposed method and seven other representative methods on the LOL and LSRW paired datasets. The results have been summarized in Table 1, which shows the average MAE, PSNR, SSIM, and LPIPS scores for these two common datasets. In terms of evaluation metrics, higher PNSR and SSIM values indicate better image quality, whereas lower MAE, LPIPS and NIQE values suggest superior image quality. Observing the table, it can be inferred that no single method can achieve the best value for all image quality detection indicators. However, our method has outperformed the others in several areas. For instance, in the LOL dataset test, our method demonstrated the best performance in the PSNR index, with LPIPS ranking second among most methods. On the other hand, in the LSRW dataset test, our method was found to perform the best in all other indicators except for LPIPS.

**Table 1:** Quantitative comparison on LOL and LSRW datasets.

Method	LOL				LSRW			
	MAE$\downarrow$	PSNR$\uparrow$	SSIM$\uparrow$	LPIPS$\downarrow$	MAE$\downarrow$	PSNR$\uparrow$	SSIM$\uparrow$	LPIPS$\downarrow$
Input	0.3914	7.7733	0.1952	0.4191	0.3202	9.0625	0.1656	0.4634
HE	0.1923	12.8575	0.3252	0.4596	0.1879	12.9180	0.3369	0.4376
Tone Mapping	0.1517	16.5034	0.5092	0.2312	*0.1179*	*17.3502*	0.4847	0.2668
RUAS	0.1534	16.4047	0.4997	**0.1937**	0.1436	15.6909	*0.4955*	0.3622
Retinex-Net	*0.1255*	*16.7740*	0.4196	0.3758	0.1185	16.8181	0.4061	0.3627
SCI	0.1912	14.7840	*0.5220*	0.2385	0.1510	15.7003	0.4387	**0.2381**
Zero-DCE	0.1860	14.7971	**0.5573**	0.2368	0.1356	16.3374	0.4739	0.2471
RRDNet	0.2739	11.4037	0.4575	0.2480	0.2002	13.3538	0.4032	*0.2469*
Ours	**0.1168**	**18.3106**	0.4876	*0.2049*	**0.0976**	**18.7179**	**0.5381**	0.3261

The best result is in bold whereas the second best results are in italic, respectively.

In addition, we also evaluated these datasets using the non-reference image quality evaluator (NIQE), as shown in Table 2. Except for Zero-DCE, which had the best score on some datasets, our NIQE scores outperformed most of the other methods. Overall, Tables 1 and 2 provide stronger evidence for the effectiveness and applicability of our proposed method.

**Table 2:** NIQE scores on low-light image sets(LOL, LSRW, LIME, MEF, NPE).

Method	LOL	LSRW	LIME	MEF	NPE	Average
HE	8.1541	3.6979	6.8883	3.5638	*3.9782*	5.2564
Tone Mapping	7.8310	*3.6757*	*3.9201*	3.5254	4.1354	4.6175
RUAS	*6.3400*	5.4509	5.3642	5.4255	7.0920	5.9345
Retinex-Net	8.8781	4.1100	4.7669	4.4097	4.6004	5.3530
SCI	7.8766	3.8157	4.2064	3.6277	4.4560	4.7965
Zero-DCE	7.7925	**3.6152**	3.9733	**3.3023**	**3.9480**	*4.5263*
RRDNet	7.4777	3.9367	4.0689	3.4796	4.0348	4.5995
Ours	**5.1504**	3.7099	**3.8218**	*3.4696*	4.2120	**4.0727**

The best result is in bold whereas the second best results are in italic, respectively. Smaller NIQE scores indicate a better quality of perceptual tendency.

## Conclusion and future work

We present a novel and effective method for solving the challenging problem of low-light images. Our approach involves using a decomposition network to obtain the illumination component map and reflection component map, followed by an enhancement network designed for reflectivity denoising and illumination enhancement. Specifically, we use RDM to denoise the reflection component, and IEM to enhance the detail and brightness of the illumination component of the low-illumination image in three iterations. Finally, we multiply the denoised reflection component and corrected illumination component elements for image reconstruction. Comparative experiments on multiple public paired/unpaired benchmarks demonstrate that our reconstructed image has uniform brightness, prominent details, small color distortion, and is true and natural from a subjective perspective. Additionally, it performs well when compared to objective evaluation indicators. However, our method still has some limitations. For images with non-uniform illumination, our method’s enhancement ability can become unbalanced, leading to potential color distortion and a subsequent loss of image integrity. This can result in the emergence of a halo phenomenon, further affecting the overall quality of the enhanced image. In future work, we plan to optimize the network structure and integrate the complete network into an end-to-end architecture.

## Data Availability

These data can be found here: LOL https://daooshee.github.io/BMVC2018website/, LSRW https://github.com/JianghaiSCU/R2RNet, LIME MEF and NPE https://drive.google.com/drive/folders/1lp6m5JE3kf3M66Dicbx5wSnvhxt90V4T.

## References

[1] Meng, L. *et al.* Adavit: Adaptive vision transformers for efficient image recognition. In *Proceedings of the IEEE/CVF Conference on Computer Vision and Pattern Recognition*, 12309–12318 (2022).

[2] Fang W, Wang L, Ren P (2019). Tinier-yolo: A real-time object detection method for constrained environments. IEEE Access.

[3] Strudel, R., Garcia, R., Laptev, I. & Schmid, C. Segmenter: Transformer for semantic segmentation. In *Proceedings of the IEEE/CVF international conference on computer vision*, 7262–7272 (2021).

[4] Fujiyoshi H, Hirakawa T, Yamashita T (2019). Deep learning-based image recognition for autonomous driving. IATSS Res..

[5] Land EH, McCann JJ (1971). Lightness and retinex theory. Josa.

[6] Wei, C., Wang, W., Yang, W. & Liu, J. Deep retinex decomposition for low-light enhancement. In *British Machine Vision Conference* (British Machine Vision Association, 2018).

[7] Zhao Z (2021). Retinexdip: A unified deep framework for low-light image enhancement. IEEE Trans. Circuits Syst. Video Technol..

[8] Liu S, Long W, He L, Li Y, Ding W (2021). Retinex-based fast algorithm for low-light image enhancement. Entropy.

[9] Hui Y, Wang J, Shi Y, Li B (2022). Low light image enhancement algorithm based on detail prediction and attention mechanism. Entropy.

[10] Zhang K, Zuo W, Chen Y, Meng D, Zhang L (2017). Beyond a Gaussian denoiser: Residual learning of deep CNN for image denoising. IEEE Trans. Image Process..

[11] Ahn, H., Keum, B., Kim, D. & Lee, H. S. Adaptive local tone mapping based on retinex for high dynamic range images. In *2013 IEEE International Conference on Consumer Electronics (ICCE)*, 153–156 (IEEE, 2013).

[12] Huang S-C, Cheng F-C, Chiu Y-S (2012). Efficient contrast enhancement using adaptive gamma correction with weighting distribution. IEEE Trans. Image Process..

[13] Stark JA (2000). Adaptive image contrast enhancement using generalizations of histogram equalization. IEEE Trans. Image Process..

[14] Guo X, Li Y, Ling H (2016). Lime: Low-light image enhancement via illumination map estimation. IEEE Trans. Image Process..

[15] Rahman S, Rahman MM, Abdullah-Al-Wadud M, Al-Quaderi GD, Shoyaib M (2016). An adaptive gamma correction for image enhancement. EURASIP J. Image Video Process..

[16] Pizer SM (1987). Adaptive histogram equalization and its variations. Comput. Vis Graph. Image Process..

[17] Reza AM (2004). Realization of the contrast limited adaptive histogram equalization (clahe) for real-time image enhancement. J. VLSI Signal Process. Syst. Signal Image Video Technol..

[18] Rahman Z (2022). Diverse image enhancer for complex underexposed image. J. Electron. Imaging.

[19] Dai Q, Pu Y-F, Rahman Z, Aamir M (2019). Fractional-order fusion model for low-light image enhancement. Symmetry.

[20] Jobson DJ, Rahman Z-U, Woodell GA (1997). Properties and performance of a center/surround retinex. IEEE Trans. Image Process..

[21] Rahman, Z.-u., Jobson, D. J. & Woodell, G. A. Multi-scale retinex for color image enhancement. In *Proceedings of 3rd IEEE international conference on image processing*, vol. 3, 1003–1006 (IEEE, 1996).

[22] Jobson DJ, Rahman Z-U, Woodell GA (1997). A multiscale retinex for bridging the gap between color images and the human observation of scenes. IEEE Trans. Image Process..

[23] Lam, E. Y. Combining gray world and retinex theory for automatic white balance in digital photography. In *Proceedings of the Ninth International Symposium on Consumer Electronics, 2005.(ISCE 2005).*, 134–139 (IEEE, 2005).

[24] Rahman Z, Pu Y-F, Aamir M, Wali S (2021). Structure revealing of low-light images using wavelet transform based on fractional-order denoising and multiscale decomposition. Vis. Comput..

[25] Lore KG, Akintayo A, Sarkar S (2017). Llnet: A deep autoencoder approach to natural low-light image enhancement. Pattern Recogn..

[26] Lv F, Lu F, Wu J, Lim C (2018). Mbllen: Low-light image/video enhancement using cnns. In BMVC.

[27] Zheng, C., Shi, D. & Shi, W. Adaptive unfolding total variation network for low-light image enhancement. In *Proceedings of the IEEE/CVF International Conference on Computer Vision (ICCV)*, 4439–4448 (2021).

[28] Wu, W. *et al.* Uretinex-net: Retinex-based deep unfolding network for low-light image enhancement. In *Proceedings of the IEEE/CVF Conference on Computer Vision and Pattern Recognition (CVPR)*, 5901–5910 (2022).

[29] Liu, R., Ma, L., Zhang, J., Fan, X. & Luo, Z. Retinex-inspired unrolling with cooperative prior architecture search for low-light image enhancement. In *Proceedings of the IEEE/CVF Conference on Computer Vision and Pattern Recognition*, 10561–10570 (2021).

[30] Guo, C. *et al.* Zero-reference deep curve estimation for low-light image enhancement. In *Proceedings of the IEEE/CVF conference on computer vision and pattern recognition*, 1780–1789 (2020).

[31] Li, C., Guo, C., Feng, R., Zhou, S. & Loy, C. C. Cudi: Curve distillation for efficient and controllable exposure adjustment. arXiv preprint arXiv:2207.14273 (2022). 10.1109/TPAMI.2026.3700822 42258694

[32] Zhu, A. *et al.* Zero-shot restoration of underexposed images via robust retinex decomposition. In *2020 IEEE International Conference on Multimedia and Expo (ICME)*, 1–6 (IEEE, 2020).

[33] Ma, L., Ma, T., Liu, R., Fan, X. & Luo, Z. Toward fast, flexible, and robust low-light image enhancement. In *Proceedings of the IEEE/CVF Conference on Computer Vision and Pattern Recognition*, 5637–5646 (2022).

[34] Hu, J., Shen, L. & Sun, G. Squeeze-and-excitation networks. In *Proceedings of the IEEE conference on computer vision and pattern recognition*, 7132–7141 (2018).

[35] Wang Z, Bovik AC, Sheikh HR, Simoncelli EP (2004). Image quality assessment: from error visibility to structural similarity. IEEE Trans. Image Process..

[36] Zhang, R., Isola, P., Efros, A. A., Shechtman, E. & Wang, O. The unreasonable effectiveness of deep features as a perceptual metric. In *Proceedings of the IEEE conference on computer vision and pattern recognition*, 586–595 (2018).

[37] Mittal A, Moorthy AK, Bovik AC (2012). No-reference image quality assessment in the spatial domain. IEEE Trans. Image Process..

